# The Impact of Volunteering Motivation for Older Korean Adults on Social Integration and Role Identity

**DOI:** 10.1093/geroni/igab046.1174

**Published:** 2021-12-17

**Authors:** Meeryoung Kim

**Affiliations:** Daegu University, Daegu, Kyongsang-bukto, Republic of Korea

## Abstract

As life expectancy increases, older Korean adults need more activities for the next 20 to 30 years after their retirement. Rowe and Kahn indicate active social participation as an area of successful aging. After retirement, older adults uphold a desire to be part of society. This study examines the motivation effects for volunteering on social integration, role identity and volunteer satisfaction. Subjects for this study are 303 older volunteers belonging to the Korean Senior Citizens’ Association throughout South Korea. According to the results, the skills obtained through volunteering had a significant effect on social integration, role identity, and volunteer satisfaction. Value motivation also had a significant effect on social integration, and reinforcement motivation significantly affected role identity. Implications of this study were found to have various effects according to the motivation for volunteering. Therefore, it will be important to understand the older adults’ motives so that they can volunteer accordingly.

